# Hardware-in-the-Loop-Based Real-Time Fault Injection Framework for Dynamic Behavior Analysis of Automotive Software Systems

**DOI:** 10.3390/s22041360

**Published:** 2022-02-10

**Authors:** Mohammad Abboush, Daniel Bamal, Christoph Knieke, Andreas Rausch

**Affiliations:** Institute for Software and Systems Engineering, Technische Universität Clausthal, 38678 Clausthal-Zellerfeld, Germany; daniel.bamal@tu-clausthal.de (D.B.); christoph.knieke@tu-clausthal.de (C.K.); andreas.rausch@tu-clausthal.de (A.R.)

**Keywords:** automotive software systems, fault injection (FI), hardware-in-the-loop (HiL), real-time, multivariate dynamic behavior, model-based development

## Abstract

A well-known challenge in the development of safety-critical systems in vehicles today is that reliability and safety assessment should be rigorously addressed and monitored. As a matter of fact, most safety problems caused by system failures can lead to serious hazards and loss of life. Notwithstanding the existence of several traditional analytical techniques used for evaluation based on specification documents, a complex design, with its multivariate dynamic behavior of automotive systems, requires an effective method for an experimental analysis of the system’s response under abnormal conditions. Simulation-based fault injection (FI) is a recently developed approach to simulate the system behavior in the presence of faults at an early stage of system development. However, in order to analyze the behavior of the system accurately, comprehensively and realistically, the real-time conditions, as well as the dynamic system model of the vehicle, should be considered. In this study, a real-time FI framework is proposed based on a hardware-in-the-loop (HiL) simulation platform and a real-time electronic control unit (ECU) prototype. The framework is modelled in the MATLAB/Simulink environment and implemented in the HiL simulation to enable the analysis process in real time during the V-cycle development process. With the objective of covering most of the potential faults, nine different types of sensor and actuator control signal faults are injected programmatically into the HiL system as single and multiple faults without changing the original system model. Besides, the model of the whole system, containing vehicle dynamics with the environment system model, is considered with complete and comprehensive behavioral characteristics. A complex gasoline engine system is used as a case study to demonstrate the capabilities and advantages of the proposed framework. Through the proposed framework, transient and permanent faults are injected in real time during the operation of the system. Finally, experimental results show the effects of single and simultaneous faults on the system performance under a faulty mode compared to the golden running mode.

## 1. Introduction

Modern automotive software systems have become heterogeneous, complex and safety-critical systems. They may consist of numerous heterogeneous components and subsystems connected to the physical components of the system. Moreover, the total number of installed embedded ECUs is growing exponentially and may include up to 120 ECUs and more than 5 system buses in modern vehicles [1]. Conversely, increasingly sophisticated functions within these systems lead to an increase in the likelihood of errors occurring. The higher the complexity of an automotive software system, the greater the probability of a fault occurrence. A malfunction in a safety-critical system, such as a steering system, can lead to catastrophic situations that affect not only human safety, but also infrastructure and the environment. Assessing the safety and reliability of sophisticated automotive systems in the early stages of the software development process is therefore a challenge in any industry. Failure mode and effects analysis (FMEA) [2] and fault tree analysis (FTA) [3] are traditionally used as analytical techniques for evaluation based on specifications and requirement documents. However, the aforementioned techniques have some limitations [4]. Such issues include the inability to represent the dynamic behavior of the system, the need for professionals with a deep understanding of the control strategy and its implementation to analyze the impact of failures on the system and, finally, the lack of sufficient tools to deal with complex systems. Therefore, test-based proofs are needed to overcome the limitations of traditional techniques, since the effects of a fault are not always clearly known in advance, especially in the development process of a complex system. Fault injection (FI) [5] is strongly recommended in the ISO 26262 standard for the automotive industry as a test method for evaluating the effects of a fault within the system, components, hardware parts or software units. During this process, the fault, error or failure is injected and the reaction at the observation points is observed [6]. From the perspective of model-based development, FI is defined as an experimentally based test method applied to the executable system model to track, analyze and evaluate the response of the system under abnormal conditions. The method has also proven useful in the area of the robustness verification of safety mechanisms, i.e., evaluating the diagnostic coverage of a safety mechanism [7]. During the development process of automotive embedded software, FI can also be used to identify the threats to functional safety according to ISO 26262 at different levels and on both sides of the classical V-model [8].

For several years, great efforts have been made to investigate model-level FI methods [9]. However, most of the previous studies do not consider the time constraints and ignore the real-time FI. Moreover, while implementing failure modes, the studies do not take into account the fact that changing the system model has an impact on the real-time characteristics of the control task, since the execution of the added blocks requires additional time [10].

Recently, the HiL platform has attracted the attention of researchers to perform the simulation in real time instead of real hardware [11]. Due to the significant features of the HiL simulation tool, research on the development of a real-time simulation platform has become very popular. The use of the tools is not limited to the verification and validation of control software, but also extends to the design and development of new applications in the automotive, aerospace and railway industries. The structure and applications of HiL simulation in dynamics and control engineering have been reviewed in [12], focusing on the design, development and implementation aspects in various domains.

In this paper, a real-time FI framework for automotive software testing is proposed. Particularly, the HiL simulation platform is used to develop and implement the proposed framework, considering the functionality of the real ECU and the whole system model with its environment. It aims to analyze the dynamic behavior of complex automotive software systems during the V-cycle development process, i.e., the system integration phase. We demonstrate the benefits and applicability of the proposed framework using a gasoline engine system as a case study, where most of the potential fault types occurring in a sensor signal have been injected in real time and the effects on system behavior have been illustrated.

The main contribution of the study is summarized below:

- The proposed framework is able to analyze the impact of the faults on the complex system in real time;
- Besides, it enables the validation of a real/soft ECU performance under nominal and faulty working conditions with complex test scenarios;
- Following that, the majority of common faults in automotive software signals are taken into account, such as gain, offset/bias, noise, hard-over, spike, stuck-at, packet loss, delay and drift faults;
- Moreover, not only single faults, but also multiple faults are simulated and injected by the proposed framework to analyze the impact of simultaneous faults on the system;
- Finally, fault modes are modeled programmatically and injected in real time via the HiL platform without the need to modify the original system model or add additional blocks.

The rest of the paper is organized as follows: Section 2 provides a background overview and discusses studies related to FI testing. In Section 3, the architecture of the real-time fault framework with the HiL simulation is presented. As a case study, Section 4 describes the system architecture, including the details of the hardware and software setup. Following this, Section 5 describes the implementation and experimental results, involving an analysis and discussion of the results obtained from the recorded experimental data sets. Finally, Section 6 presents the conclusions and future work.

## 2. Background and Related Works

In this section, a brief overview of the background of the topics covered is presented. In addition, the proposed methods and tools for FI during the development process are discussed based on the current state-of-the-art.

### 2.1. Fault Injection Approach

Fault injection is a system reliability assessment technique in which faults are intentionally introduced into a system through controlled experiments and the behavior of the system in the presence of faults is observed [13]. This approach enables an effective evaluation of various dependability attributes, i.e., safety, reliability, availability and security against random faults [14,15]. Most of the faults considered in this approach include nonlinear and unpredictable faults, called random hardware (HW) faults, which are mainly caused by environmental factors and occur as degradation of component functionality. An example of a random HW fault is a delay or loss of data in a communication malfunction. In contrast, systematic faults are deterministic and are caused by design-related factors, such as deviations in device specifications.

FI methods are commonly used to evaluate fault-tolerant systems, i.e., to assess the robustness of fault tolerance in the presence of faults [16,17]. Compared to the other analytical methods, this method differs in that the faults are injected into the system and then the dynamic behavior of the system is observed and analyzed experimentally. Typically, a FI environment consists of five main components, namely, controller, fault injector, system/concept under test, load generator and monitor [5]. The fault load library and work load library should also be defined in advance as inputs to the injector and load generator, respectively. To this end, the inputs and faults to be injected into the target system are defined either based on expert knowledge or using probability distributions. The key attributes of FI comprise a set of faults (F) with which the injection is to be carried out, activations (A) practiced at the time of the experiment, a set of readouts (R) representing the output domain and derived evaluation measures (M) resulting from applying fault test cases, which, together, form the FARM environment model [16].

In the literature, several FI strategies have been proposed. In accordance with the method on which FI is based, they can be classified into five categories, namely, hardware-based, software-based, simulation-based, emulation-based and hybrid FI. Extensive information about the advantages and disadvantages of each technique can be found in [13]. A variety of techniques for performing each method have been proposed as a result. For example, code change injection, data FI and interface FI are the main techniques for implementing software-based FI [18]. Some tools presented in the literature based on these techniques are XCEPTION [19], FIAT [20], FTAPE [21], Orchestra [22] and FERRARI [23]. On the other hand, simulator commands, saboteurs and mutant-based techniques are used to perform simulation-based FI [24]. VERIFY [25], MEFISTO [26] and SST [27] are some tools proposed depending on simulation-based FI.

### 2.2. Model-Based System Testing Phases

**Figure 1:**
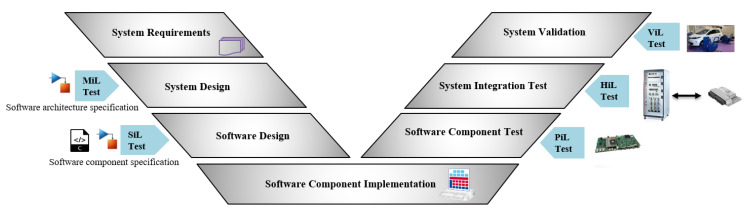
V-cycle of model-based software development process with the test phases.

Model-based development is a powerful approach used in many areas of the automotive industry for the development of software systems, especially for the design and implementation of complex embedded systems. As it enables verification and validation at very early stages of the development process, it plays an important role in reducing the development time and saves significant development cost and effort [28]. Moreover, in recent years, there has been a growing interest in the automatic generation of vehicle software code from the model. Nevertheless, various test phases should be performed during the system’s life cycle for ensuring the quality of the generated code and detecting unexpected defects. The V-model, which contains the various test phases and design processes in the model-based development approach, is shown in Figure 1. Two sides make up the model: The design phase on the left side and verification and validation (V&V) on the right side. The left side allows two different test phases to be performed without physical hardware components, namely, modeling-in-the-loop (MiL) [29] and software-in-the-loop (SiL) [30]. The right-hand side, on the other hand, contains three test phases, namely, processor-in-the-loop (PiL) [31], hardware-in-the-loop (HiL) [11] and vehicle-in-the-loop (ViL) [32].

By enabling a system to be developed, modified and tested quickly, MiL offers an important advantage in terms of early testability in the test environment. In this process, the functional behavior of the system is verified by simulating the controller, as well as the plant, in a simulation environment. MATLAB/Simulink [33] from mathwork and TargetLink [34] from dSPACE are considered to be among the most useful tools in the field of modeling and simulation. Thanks to the aforementioned tools, the system to be developed can be modeled and simulated at a very early stage of the development process. In addition, for the verification and validation of the system functionality, the system model can be efficiently executed.

The next phase on the left side of the V-model is SiL, where the correctness of the code generated from the system model is verified in a virtual environment without hardware. The generated code of the controller model, i.e., the S-function block, is connected to the system model and tested with different stimuli. In this process, not only is the performance of the controller analyzed and debugged, but also the production code is tested in a cost-efficient way.

PiL on the other side of the V-model represents the first phase for verifying the behavior of the control system in the real prototype, in which, the generated production code is implemented in the embedded target processor, e.g., ECU. During this phase, the plant model interacts directly with the real target machine, except that real-time constraints are not taken into account.

Once the production code is verified on the target machine, the next test phase is to validate the performance of the control system in real time. Toward this end, the plant model code is generated and fed into the hardware simulator. By this means, the plant model directly communicates with the real physical control system in real time and performs HiL. In this study, the developed FI framework is implemented in real-time using the HiL simulator connected with a real-time system (MicroAutoBox II) via CAN bus. Therefore, details of the HiL test phase with its components and configuration are presented.

### 2.3. Fault Injection in System Development Life Cycle

FI is a powerful technique used in the different phases of the system development process to verify and validate safety and reliability aspects. This technique is based on the idea of injecting faults into the system under test (SUT), allowing its response to abnormal conditions to be analyzed and evaluated. FI is strongly recommended by the functional safety standard ISO 26262 [35]. As demonstrated by Pintard et al. [8], the FI method can be used on both sides of the ISO 26262 V-cycle.

#### 2.3.1. MiL-Based Fault Injection

Based on a system model that simulates the behavior of a controlled system with a control system, model-based techniques have attracted much attention from academic and industrial researchers to analyze, verify and validate system behavior in the early stage of system development [36]. One of the first examples of using models to simulate hardware fault behavior, based on the FI approach, is presented in [37,38], where the executable model of the system was executed using existing commercial tools, e.g., Simulink or SCADE. To combine the advantages of hardware-based FI and software-based FI, Moradi et al. [10] proposed model-implemented hybrid tools using Simulink as the modeling environment. However, the proposed framework is limited to injecting a single fault per model. In addition to this, to ensure the real-time behavior of the system, slack parameters are required as further manual steps to determine the slack time and the number of additional blocks to be added to the system model. Our framework allows for the injection of both single and multiple faults, enabling mixing and simultaneous faults to be activated. Moreover, the faults are injected programmatically without modification of the original system model.

In the automotive domain, some authors have developed FI techniques to be used to verify safety objectives or safety mechanisms in accordance with ISO 26262 in early design phases [39,40]. In 2019, Saraoglu et al. presented a FI-based testing simulation framework called MOBATsim implemented on Simulink behavioral models [41]. This framework allows for the injection of hardware faults into the model to evaluate the safety of autonomous driving systems at different levels, i.e., component, vehicle and traffic levels. However, the proposed simulation framework is designed at the simulation level without considering the real-time characteristics of the control task. Our FI framework, however, is implemented in the real-time simulation platform, enabling precise analysis in real time. Similarly, Juez et al. [42] investigated the applicability of a simulation-based FI framework called Sabotage using the vehicle simulator dyanacar. The focus of the proposed work was to determine the most appropriate safety concept and early safety assessment of the lateral control system of a vehicle according to ISO 26262 at the simulation level. Although the authors considered the whole vehicle system for safety analysis, model blocks are added to the system model to represent failure modes, which is not effective in a complex system and results in violating the real-time system behavior. Our proposed framework is based on programmatically manipulating the sensors signals while ensuring real-time properties.

In addition to the above mentioned works, several publications have appeared in recent years documenting model-based FI tools in the area of a safety and reliability assessment of automotive software systems, such as Kayotee [43], ErrorSim [44], AVFI [45], FIEEV [46], SIMULTATE [47] and EQUITAS [28]. Although there are many studies focusing on the development of FI methods and tools at the simulation level for various domains, there are many problems in the existing research in representing the proper effects of faults considering real-time constraints. However, in our study, we used a real-time simulator with a real-time control system to develop our proposed framework, offering high fault coverage along with high fidelity simulations for complex system behavior analysis.

#### 2.3.2. HiL-Based Fault Injection

Despite the fact that the HiL simulation has been traditionally used for the design and development of new ECUs in the automotive industry [11], academic scholars have made great efforts to investigate the development of automotive control software based on the HiL platform. For example, Palladino et al. proposed a portable electronic environmental system called a micro-HiL system [48]. It aims to evaluate the engine control software strategies and diagnose its functions on a CAN bus utilizing the 1.6-liter Fiat gasoline engine as a case study. In [49], a new concept for the development of advanced driver assistance systems is proposed based on vehicle HiL simulation. In the railway field, Conti et al. [50] investigated the analysis of a railway braking system under degraded adhesion conditions based on a HiL approach, highlighting the advantages of the proposed approach in terms of both the testing cost and reproducibility, especially for analyzing the system behavior under good and degraded adhesion conditions during the braking of a railway vehicle.

Along with the advances in the HiL real-time simulation for embedded control development and automated testing, an analysis of complex software systems’ behavior under abnormal conditions has attracted much attention in the last decade. Several methods addressing this issue have been described in the literature. For example, Poon et al. [51] have conducted a study to demonstrate the capability of a HiL platform with FI in testing electrical vehicle drive systems. Three different operating and fault conditions are used in the proposed study to validate the fidelity of the real-time simulation, where the real drive system of an electric vehicle and the real-time simulation have been compared. However, FI in this study is limited to specific fault modes in the drive systems and is employed to validate the fidelity of the proposed HiL platform, but our study focuses on the development and design of an effective real-time FI framework with high fault coverage for complex software systems analysis. Yang et al. [52] proposed a multiprocessor HiL FI strategy that aims to simulate various faults in the traction control system (TCS). In the proposed platform, three fault scenarios are used for real-time FI in the HiL simulation, i.e., an open-switch fault of the power transistor, a stuck fault of the three-phase current sensor and a broken rotor bar fault of the traction motor. Although the proposed FI method is developed using a physical traction control unit (TCU) and a real-time simulator, the FI unit is designed in FPGA based on the logical operators to satisfy the time constraint, which leads to an increase in the manual effort in terms of the injection point in a complex system. However, to inject the faults, our framework is based on manipulating the signals accessed on the CAN bus in accordance with the user’s specifications in terms of the location, time and type. Concerning the same area, to evaluate the risks in railway traction drive and to analyze its behavior, an improvement of FMEA using a HiL-based FI approach was proposed in [53]. In the proposed research, the focus was on improving the FMEA methodology to provide a quantitative analysis using FI with the purpose of creating failure scenarios. However, to implement the failure modes, the system model was extended, which, in turn, affects the real-time system behavior; however, in our framework, this issue has been addressed by treating both the plant model and the control system model as a black box without modification. In the context of automotive sensor networks, Elgharbawy et al. proposed a real-time functional robustness verification framework for multi-sensor data fusion algorithms applied to radar and camera sensors in advanced driver assistance systems (ADAS) [54]. HiL co-simulation with run-time-implemented FI has been used to simulate sensor faults involving latency, detection errors and false one-to-many object labeling. However, though the conducted study is limited to the investigation of certain critical driving situations by focusing only on imaging sensors and range sensors to verify the robustness of the fusion algorithms, in our framework, all sensors signals accessed via the CAN bus can be manipulated, which increases the fault locations coverage for analysis objectives.

Online condition monitoring and fault diagnosis in a real-time environment based on the HiL simulation are other areas where the FI approach can be used. For example, recent research in [55] proposes that a short-circuit FI model can be used to realize online switching between healthy and faulty states of induction motors, with the aim of determining the trend of the change in fault characteristics, as well as the fault level. Although the fault source modeling in the proposed work reduces the modeling effort, it is limited to one fault mode, i.e., the stator interturn short-circuit fault, which is activated by changing the induction motor parameters and switching between the operating states. However, in our proposed framework, not only the healthy state but also nine different fault types that can be injected as a faulty state in the online simulation can be realized. Garramiola et al. [56] have used the FI approach to develop a hybrid sensor fault diagnosis methodology in railway traction drives using the HiL platform. This is accomplished by injecting gain and offset sensor faults into the DC link voltage and catenary current sensor using a FI signal so that the dynamic response and robustness under fluctuations, as well as the sensitivity of the fault reconstructions, can be analyzed. In the mentioned study, FI has been investigated from the point of view of developing and verifying a fault diagnosis system; therefore, the application is different. However, our study focuses on the development of real-time FI as a testing method during the system development phases.

Safety verification during design at the component and system levels is of growing interest in the automotive industry, as it is critical for confirming safety properties and identifying safety faults. To address this issue, many researchers have proposed FI frameworks and tools as a measure to verify vehicle functional safety. For example, a retargetable vehicle-level FI framework capable of automatically injecting various faults into the processor, memory or IO at the runtime was proposed in [57]. The proposed framework has been validated and demonstrated using an experimental HIL test for autonomous driving, i.e., EcoTwin truck platooning. Compared to our proposed framework, this framework was developed using a software-based FI approach, whereas our framework relies on signal modification as the basis for FI. In addition, the target components of the SUT in this study are processor registers, memory, IO and OS kernel, but, in our framework, the sensors and control signals of actuators are the target components for FI. Park et al. [58] have also proposed a FI method for software (SW) unit/integration testing during the ECU software development process of automotive open system architecture (AUTOSAR)-based automotive software. Potential software faults in AUTOSAR-based automotive software, such as data, program flow, access, asymmetric and timing faults, were defined in the proposed study, and injected using the proposed tool. The applicability and performance of the proposed method have been demonstrated utilizing a set of actual automotive software, and the results were compared with other FI tools. Although the proposed research analyzes and compares various aspects of FI testing in the SW unit/integration testing phase, significant differences from our proposed framework exist. They developed the method for SW unit/integration testing phase focusing on software faults, whereas our proposed framework is developed to be used in the system integration testing phase during the development process. In addition, our proposed framework enables the injection of hardware faults that occur in the sensor and actuator control signals. Moreover, in our study, the entire vehicle system model has been considered to enable effective and precise testing at the system level. An overview of the related works is given in Table 1. It includes the FI approach used, the application domain, the ability to inject multiple faults, the number of fault types injected, real-time constraints consideration and the assessment in terms of manual effort, fault coverage and fidelity simulations.

According to the above observations in the previous works, the majority of the proposed research is limited to the adaptation of the FI approach for specific objectives, focusing on certain operation and fault conditions. Additionally, the development of experimentally based test methods for the dynamic behavior analysis of automotive software systems during a system integration testing phase of the V-Model has not been well explored. Specifically, a real-time FI method capable of covering a wide range of potential sensor faults in the vehicle system and considering the whole system model. Therefore, this study attempts to fill this gap in the literature by proposing a HiL-based FI framework toward analyzing the effects of faults on the automotive system in real time during the deployment process.

## 3. Methods

### 3.1. HiL-Based Real-Time Fault Injection Framework

**Figure 2:**
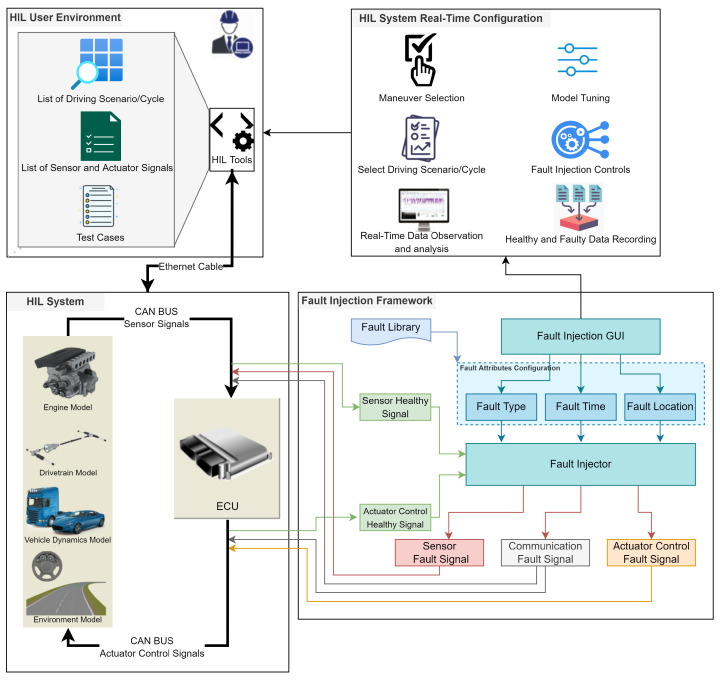
HiL-based real-time fault injection framework.

The proposed real-time FI framework consists of four main components, namely, HiL user environment, HiL system real-time configuration, HiL system and the FI framework, as shown in Figure 2.

#### 3.1.1. HiL User Environment

In HiL user environment, the tester can analyze recorded data and control HiL system in real time using HiL tools. Besides, the target system can be tested based on test cases with the list of sensors, actuator control signals and driving scenarios. HiL software tools can control HiL system from a computer machine. These tools are capable of configuring HiL system in a real-time environment. Generally, a computer and HiL system is connected with an Ethernet cable to each other for controlling and transmitting data.

#### 3.1.2. HiL System Real-Time Configuration

The HiL system real-time configuration is responsible for various controls in run time, including: Selection of maneuver and driving scenarios, model tuning, real-time data observation and analysis, data logging and controls of the FI. For logging data, the tester can also record healthy and faulty data using HiL tools in the user environment. The tester can also select the signals to be recorded in the recording configuration. The driving maneuver can be selected in the user environment. Generally, there are two options for selecting the maneuver mode: online driving and autonomous driving based on the driving cycle. In online driving, the tester can manually control some input values, such as clutch, brake, acceleration and gear, in the computer system, whereas, in autonomous driving, these values are already defined in the driving cycle.

#### 3.1.3. Fault Injection Framework

The main objective of the FI GUI is to support the tester in the HiL user environment during run-time FI. From the GUI, a tester can perform various actions. Some of them are: Selecting the fault type from the fault library and the fault values associated with the selected fault type.

In order to implement the FI method, three main configuration fault dimensions must be defined, i.e., fault type, fault location and injection time, which, together, form a fault space. According to the literature, there are several types of potential faults in the time series data, such as gain, offset/bias, noise, hard-over, spike, stuck-at, packet loss, delay and drift faults [59,60,61]. By analyzing the signal features that characterize automotive fault behavior, the authors of [62] categorized the common faults in the vehicle’s PCM signals, such as abnormal magnitudes, rolling, noise and dependency faults. In addition, they also named some direct and indirect causes of the defined faults. Dirty or deteriorated sensors, faulty vehicle components, vibrations caused by dangerous internal motors, and sudden acceleration are good examples of direct sources that can cause these types of faults. Conversely, bumpy roads and uncertainties in driving behavior can cause anomalies in signal behavior without necessarily provoking faults.

Equation (1) and Table 2 are used to summarize the mathematical representation of the aforementioned types:

(1) $$f\left(t\right)=d_{v}h\left(t\right)+o_{v}$$

where f(t) is faulty or manipulated signal value, $d_{v}$ represents the gain value and h(t) is the healthy or standard signal value. $o_{v}$ represents the offset/bias value.

**Figure 3:**
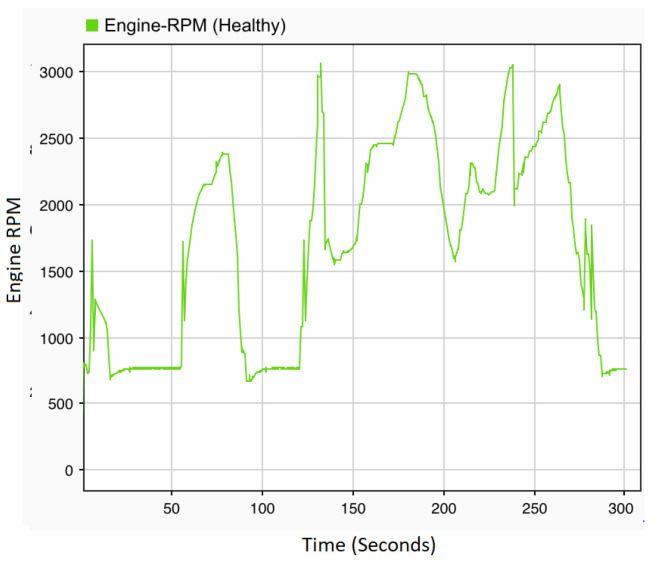
Fault-free/healthy signal.

**Figure 4:**
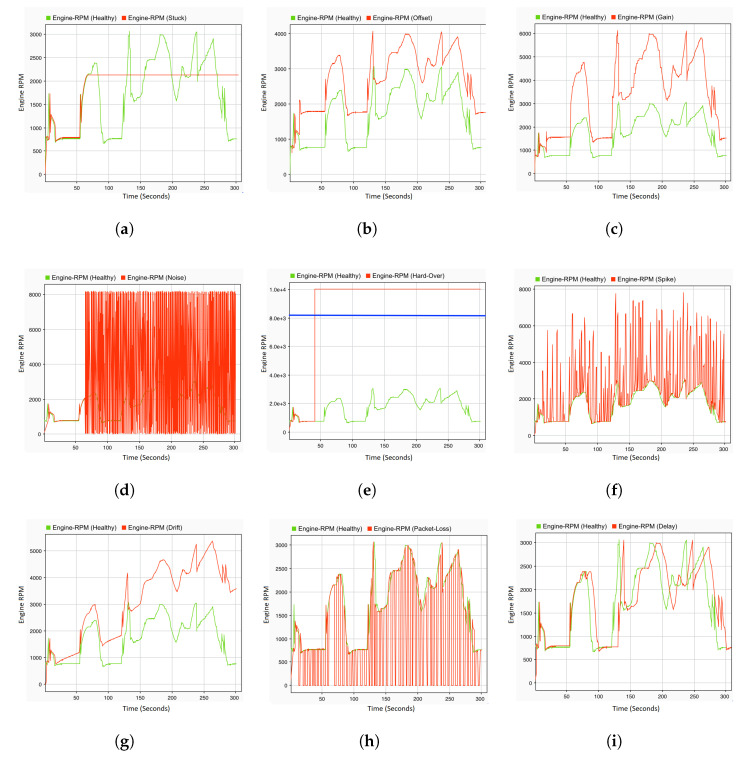
Fault types. (**a**) Stuck-at fault, (**b**) Offset fault, (**c**) Gain fault, (**d**) Noise fault, (**e**) Hard-over fault with maximum threshold, (**f**) Spike fault, (**g**) Drift fault, (**h**) Packet loss fault, (**i**) Delay fault.

Based on the healthy signal (golden run) presented in Figure 3, all types of fault defined in Table 1 are illustrated in Figure 4.

The FI time at which the fault is to be injected can be defined on the basis of the driving cycle. The duration of the occurrence of the fault can be used to distinguish between permanent/persistent faults and transient faults [63]. Transient faults are short-term faults that occur for a certain duration and disappear again after a short time. In contrast, permanent faults occur over a longer period of time and remain until fault correction is accomplished.

On the other hand, the fault location indicates the place where the fault is to be injected, e.g., the fault within an element, function or communication between components and the subsystem. The primary potential points of the fault in the vehicle have been explored in [64]. The authors classified the potential fault locations in an in-vehicle network into function specifications, network, sensors, actuators, control devices, gateways, a power supply, vehicle subsystems and a data acquisition system. In this study, the scope of fault location will cover the potential location of fault occurrence in the closed loop system, i.e., sensors, the control signal and the communication between them.

#### 3.1.4. HiL System

**Figure 5:**
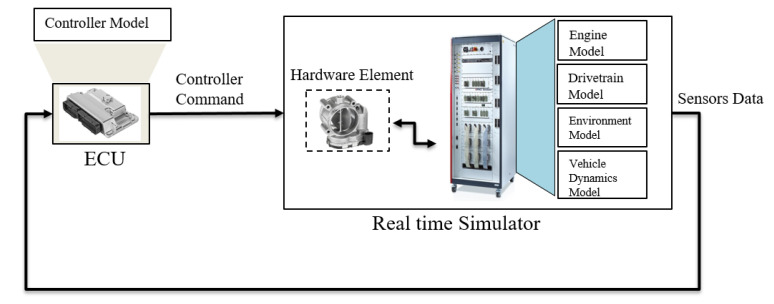
HiL real-time simulation with real ECU.

Since the HiL simulation enables an efficient, fast, realistic and highly accurate simulation with repeatability, it is widely used for the verification and validation of real-time embedded systems in various domains, such as automotive, railway and aerospace industries. The two major constituents of the HiL simulation platform are a real-time simulator and a real ECU. The HiL simulator is connected to the physical ECU via the CAN bus so that the embedded real-time control system interacts with the mathematical models simulated and deployed in the HiL simulator (see Figure 5).

In some cases, real vehicle components, such as sensors, actuators, the steering wheel and pedals, can also be connected to the HiL simulator via electrical interfaces. The control model and plant model are developed in the simulation level using the model-based development methodology. The well-known tools Simulink and SCADE are common tools for the design, simulation and verification of software systems in the early stage of the development process. Utilizing the real sensors connected to the HiL simulator or simulated sensor model, the real controller takes sensor data from various sources and sends the actuation command to the simulated actuator in the HiL simulator so that the action is performed according to the control logic. Software tools on a host PC connected to the HiL simulator via the Ethernet bus are used to set up the HiL test bench.

Real-time FI has been proposed by us to simulate various anomalies or faults in the overall system and to analyze their effects at the vehicle level, taking into account the real-time constraints. In this way, not only sensor faults but also control and communication faults can be simulated. Besides, the robustness of the safety mechanism can also be evaluated in real time. The behavior of the system under test SUT in the presence of faults can be accurately analyzed by considering vehicle dynamics and incorporating models of the environment. To be specific, the controller model is deployed and executed in the real ECU, and the dynamic models of the engine, transmission, environment and vehicle dynamics are executed on real-time processor hardware. Moreover, by enabling and selecting different driving and fault scenarios, a comprehensive validation of the SUT with a controlled simulated system under abnormal conditions can be achieved.

## 4. Case Study and Implementation: ASM Gasoline Engine

### 4.1. System Architecture and Implementation

**Figure 6:**
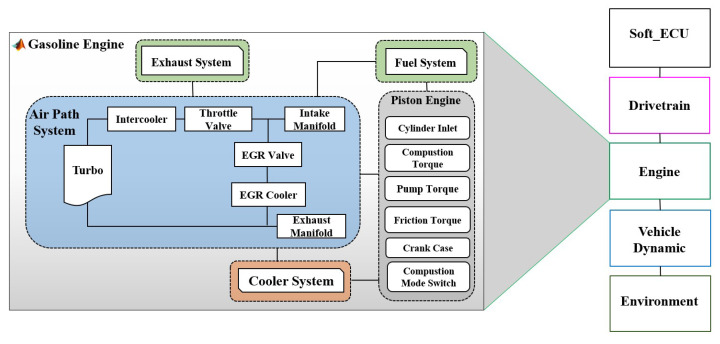
System architecture of the gasoline engine.

To demonstrate the applicability of the proposed framework, the ASM gasoline engine model from dSPACE [65] is used as a case study. Figure 6 shows the system architecture of the gasoline engine designed in the MATLAB/Simulink environment. It consists of several systems and subsystems, i.e., SoftECU, gasoline engine, powertrain, vehicle dynamics and environment. In addition, there are I/O blocks for simulation and management of signals to the real hardware ECU. SoftECU provides the simulation of controller algorithms. Thus, the plant model can either contact the real target ECU (online mode) or receive controller commands from SoftECU at the simulation level (offline mode). The gasoline engine has been modeled in detail to include all physical characteristics of the engine. Air path system, fuel system, piston engine system, exhaust system and cooler system are the main parts.

To define driving maneuvers and set environmental conditions for the vehicle, the vehicle environment and driver model are used to complement the virtual powertrain. More specifically, both the powertrain and vehicle dynamics are modeled to provide longitudinal driving characteristics, including vehicle resistances, transmission model, vehicle resistances and driver characteristics. More detailed information can be found in [65]

**Figure 7:**
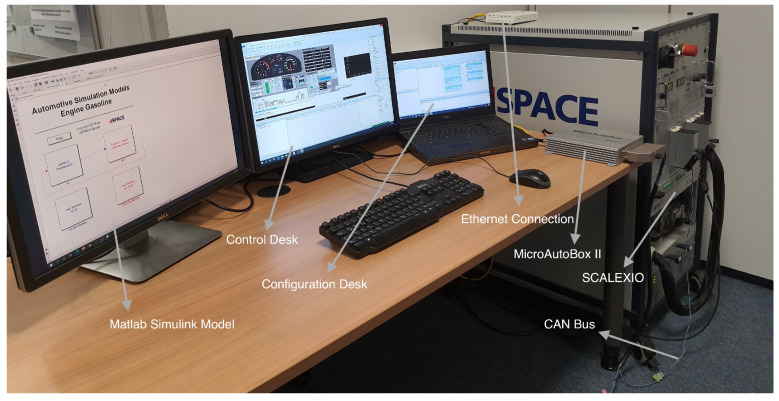
Scheme of the complete HiL simulation system.

The connection between the ECU and HiL real-time simulation system is shown in Figure 7. The ECU is used to control fuel metering and pressure control for the common rail, fuel injection quantity, injection timing, injection angles and various other control signals. In this study, dSPACE MicroAutoBox II is employed to act as a real ECU and is connected via the CAN bus to dSPACE SCALEXIO, which transmits corresponding sensor and actuator signals.

**Figure 8:**
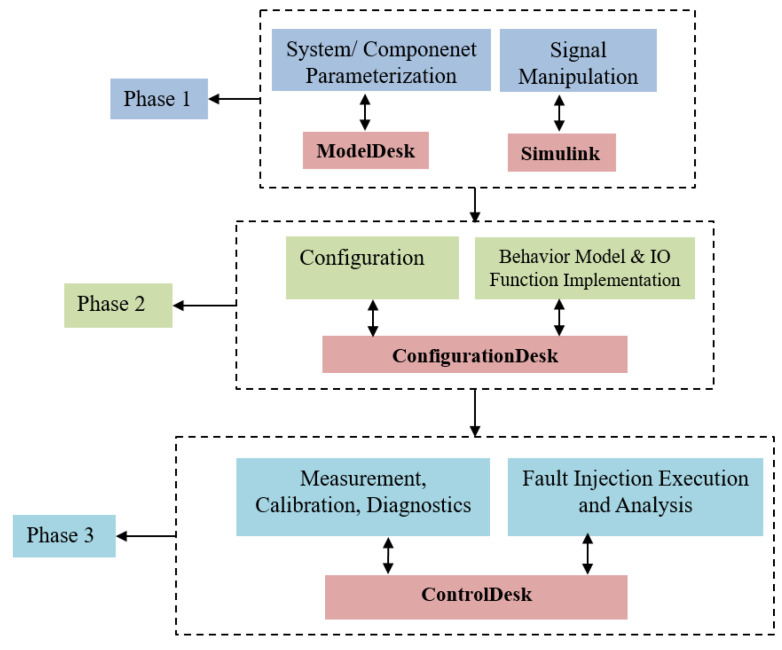
Implementation workflow of the FI framework.

The implementation of the real-time FI framework in the HiL system is carried out in three phases, namely, the modeling, configuration and control phases, as shown in Figure 8. To this end, software tools are used in each phase: Simulink and dSPACE ModelDesk in phase 1, dSPACE ConfigurationDesk in phase 2 and dSPACE ControlDesk in phase 3. The implementation workflow starts with MATLAB/Simulink, where the modeling of fault modes is implemented in the real-time interface CAN multimessage blockset (RTICANMM). This allows CAN communication network signals to be accessed and configured during the control phase. By doing so, the list of all sensors and control actuator signals can be explored with the bus navigator, and then, based on the configuration of the fault injector, the signals of the selected system model can be manipulated in real time with the ControlDesk tool. On the other hand, ModelDesk is used to parameterize the selected system with its components. Thanks to ConfigurationDesk, the variable description file containing the configurations of the ASM gasoline engine is created in phase 2. Once the variable description file has been created, the software code is automatically generated from the models. Subsequently, the generated code of both the controlled system and the controller is deployed into the target hardware, i.e., the HiL simulator and the MicroAutoBox II, respectively. Finally, the control station in Phase 3 is the place where the user can configure the experiments, including instrumentation, online parameterization with access to simulation platforms and connected bus systems, controller calibration and diagnostics and measurement execution. In addition, the faults implemented in the RTICANMM model can be accessed in the control panel via the variable control bar and manipulated in real time according to the fault configuration.

### 4.2. Setup

In the case study, several sensor signals are available, such as the crank angle sensor, battery voltage, accelerator pedal position, ignition and starter demand, EGR mass flow, engine speed, intake and exhaust manifold pressure, fuel pressure, coolant temperature and railbar. The selected actuator control signals, on the other hand, are the control of the fuel metering unit per cylinder, the injection angle per cylinder, the injection time for direct injection and the control switches. Total information about the selected location of fault occurrence is shown in Table 3

**Figure 9:**
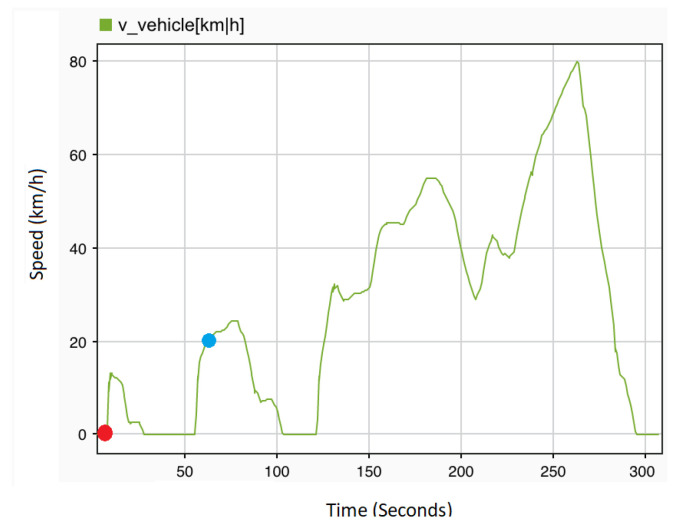
Driving cycle in dSPACE ControlDesk.

To configure the driving scenario, the driving cycle is selected from the list provided by dSPACE in controlDesk. The driving mode is set to automatic with the predefined vehicle speed. Figure 9 shows the selected drive cycles in dSPACE ControlDesk. The red dot represents the starting point and the blue dot the current location. The cycles can be changed according to the tester’s requirements. Key specifications for the HiL experiments in order to perform the aforementioned driving scenario are given in Table 4.

In this study, the effects of permanent and transient faults are demonstrated. In the context of a permanent fault, single and multiple faults are injected based on a predefined configuration. The configuration of the permanent single and multiple fault injector, including the main FI attributes, i.e., the fault location, fault type and fault time, are shown in Table 5 and Table 6, respectively. On the other hand, Table 7 lists the configuration of the transient faults.

## 5. Results and Discussion

This section discusses the results for employing the real-time FI framework proposed in this paper. The selected faults are injected based on the duration of their occurrence as permanent (a single and simultaneous/concurrent faults) and transient faults in different modes so that the effects of the faults on the system behavior can be analyzed in a real-time simulation.

### 5.1. Real-Time Permanent Fault Injection

Due to several causes, faults can occur permanently, and, in most cases, they still exist until recovery is carried out. In this paper, single and multiple permanent faults have been injected in real time using a developed FI framework.

#### 5.1.1. Single Fault Injection

**Figure 10:**
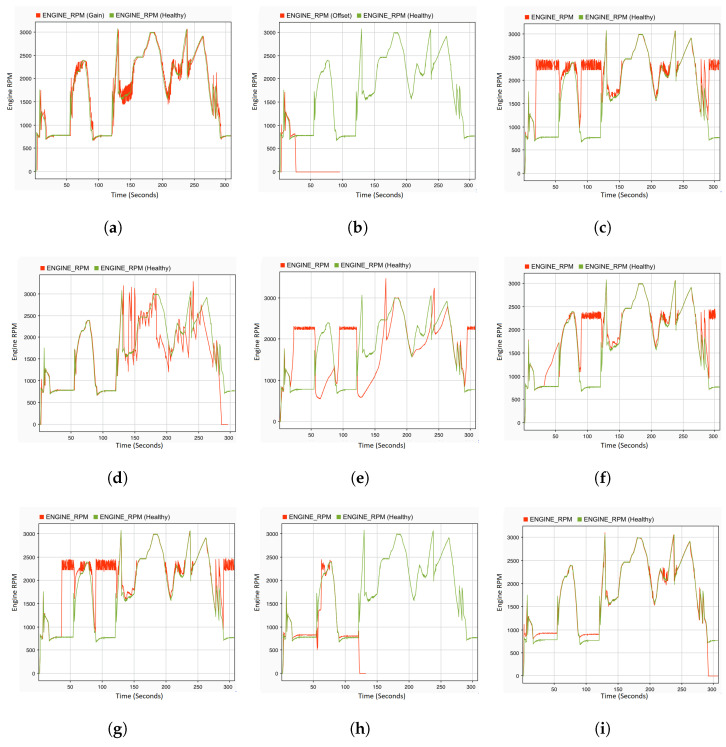
System output: engine RPM with single permanent fault injection. (**a**) Acceleration pedal with gain fault. (**b**) Engine RPM sensor with offset fault. (**c**) Acceleration pedal with noise fault. (**d**) Rail bar sensor with packet loss fault. (**e**) Engine RPM sensor with stuck-at fault. (**f**) Acceleration pedal with drift fault. (**g**) Acceleration pedal with hard-over fault. (**h**) Mass flow through throttle with spike fault. (**i**) Engine RPM sensor with delay fault.

**Figure 11:**
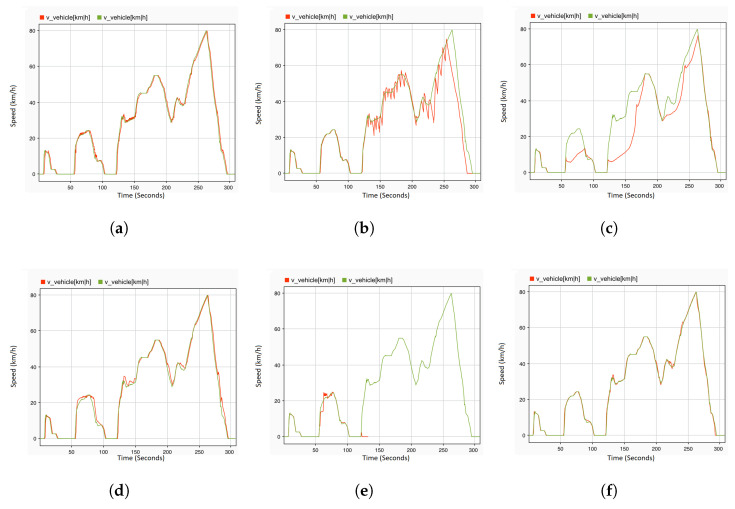
System output: vehicle speed under single permanent fault injection. (**a**) Acceleration pedal with gain fault. (**b**) Rail bar sensor packet with loss fault. (**c**) Engine RPM sensor with stuck-at fault. (**d**) Acceleration pedal with drift fault. (**e**) Mass flow through throttle with spike fault. (**f**) Engine RPM sensor with delay fault.

A single fault denotes that only a single variable is manipulated and forced to its extreme value. For the purpose of observing the reaction of the system in the presence of faults in the sensors and control signals of the actuators, all of the faults defined in Table 5 are injected in the corresponding location at the specified time. To this end, an acceleration pedal position sensor is selected as a fault location. There are various types of faults that are injected on this sensor at different times of the driving scenario. The gain fault is injected on this sensor at 5 s with $d_{v}$ a value of 10 in Equation (1). The results are recorded at system level, where the engine RPM (Figure 10a) and vehicle speed (Figure 11a) are observed. Whenever the driver tries to maintain the speed of the vehicle by making a small fluctuation in the position of the acceleration pedal, this small fluctuation results in noise in the vehicle speed and engine RPM. This noise can be seen at a time of 135 s to 145 s, and many more similar situations can be observed in Figure 10a and Figure 11a. The noise fault is also injected at 5 s of the driving scenario, when the vehicle speed is 0 and the gear knob is in a neutral position. As soon as the fault is injected, the engine RPM values on the system level fluctuate between 2200 and 2500 rpm, whereas, in a normal case (fault-free), it fluctuates between 700 and 800 rpm. This results in a higher energy consumption. Moreover, when the driver tries to maintain the speed of the vehicle, some erratic noise is observed in the engine RPM. Figure 10c shows the effect of the noise fault on the engine RPM. On the other hand, there is a very small effect observed on the vehicle speed after the injection of the noise fault. The drift fault is injected at the starting point of the driving scenario. The effects of this fault is observed on the system level values of the engine RPM (Figure 10f) from 30 s, and when the gear knob of the engine is at a neutral position. As shown in Figure 10f, the engine RPM values slowly start to increase. Moreover, there is a slight increase in the vehicle speed also observed at 85 s to 90 s and between 130 s and 150 s, as shown in Figure 11d. The hard-over fault (Figure 10g) is injected at 36 s, and the effects of this fault are immediately observed on the system level values of the engine RPM. The values immediately jump from 700–800 rpm to 2200–2500 rpm. The system values of the hard-over fault are very similar to the noise and drift fault at a time of 90 s to 120 s; however, there is a small difference. The engine RPM values after the drift fault are between 2200 and 2400 rpm, whereas they reach up to 2500 rpm in the hard-over and noise faults. Moreover, there is more noise in the engine rpm values with the hard-over fault when compared to noise fault. Up to a certain period of time, it is difficult to differentiate between these faults, e.g., after 290 s, it is difficult to determine the nature of the fault by observing the engine speed values.

To analyze the effect of the potential fault in the engine RPM sensor on the system behavior, offset and stuck-at faults are injected at 24 s and 5 s of the driving scenario, respectively. When the vehicle gear knob is at neutral position and the vehicle speed is 0, an offset fault is injected with a $o_{v}$ value of 1800 in Equation (1). As can be seen in Figure 10b, within one second of fault occurrence, the engine immediately stops and the value of the engine RPM becomes 0. In the same context, the stuck-at fault is also injected with $d_{v}$ and $o_{v}$ values of 0 in Equation (1). At the time of injection, the engine is running with a vehicle speed of approx 10 km/h. The effect of this fault is not observed at that moment, but once the engine comes into the neutral position, the engine RPM value increases to 2400 rpm at the system level. Additionally, when the vehicle starts to accelerate at 50 s, a delay in the behavior of both signals RPM and speed can be observed, as shown in Figure 10e and Figure 11c.

The delay and packet loss fault are also considered in this study as a type of communication fault category. Therefore, the delay fault is injected into the engine RPM sensor at 120 s. A very small effect can be observed on both system level values of the engine RPM and vehicle speed. As shown in Figure 10i, between 210 s and 240 s, there is a slight noise observed in the engine RPM, and its effect can be seen in the vehicle speed (Figure 11f) as well. The packet loss fault is injected into the rail bar sensor at the time of acceleration, i.e., 120 s. Figure 10d and Figure 11b demonstrate significant fluctuations and jerks in the engine speed or vehicle speed signals. Due to the fact that the time taken in the loss of packets is not more than 2 s for the whole driving scenario, the packets were lost for 3 s, which results in complete failure where the engine stopped working immediately. At the mass flow through the throttle, the sensor spike fault is injected at the starting point of the driving scenario. Once the vehicle starts to accelerate at 55–60 s, a slight downfall in the engine RPM (Figure 10h) can be observed, whereas no significant effect in vehicle speed can be observed, except for a sharp increase at 58 s and 62 s (Figure 11e). From 90 s to 120 s, there is no change in the engine RPM, but as soon as the vehicle accelerates, the engine stops immediately. At 121 s, the system level values of the engine RPM and speed are both 0.

#### 5.1.2. Simultaneous/Concurrent Faults Injection

**Figure 12:**
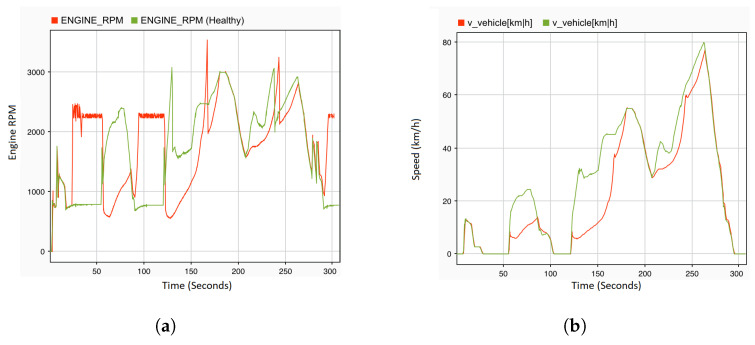
System output: engine RPM and vehicle speed under multiple permanent faults injection. (**a**) Engine RPM with stuck-at fault and noise fault. (**b**) Vehicle speed with stuck-at fault and noise fault.

The FI framework also allows for multiple faults to be injected into the sensor signals and the actuator control signals. In order to do so, more than one variable is manipulated and assigned with extreme values in such a way that the effect of the combination of several independent faults can be analyzed. In this study, two different types of faults are injected into the sensor signals at different locations and times. The noise fault is injected into the acceleration pedal sensor at 25 s and the stuck-at fault with $d_{v}$ and $o_{v}$ values of 0 (1) is injected into the engine RPM sensor at 35 s. The effect of the noise fault is observed in the first 10 s between 25–35 s of the driving scenario. Right after, the effect of both faults in the engine RPM is observed, as can be seen in Figure 12a. Looking at the speed of the vehicle in Figure 12b, it is clear that the effects of injected faults are exactly the same as the effects of a stuck fault in the engine RPM sensor.

**Figure 13:**
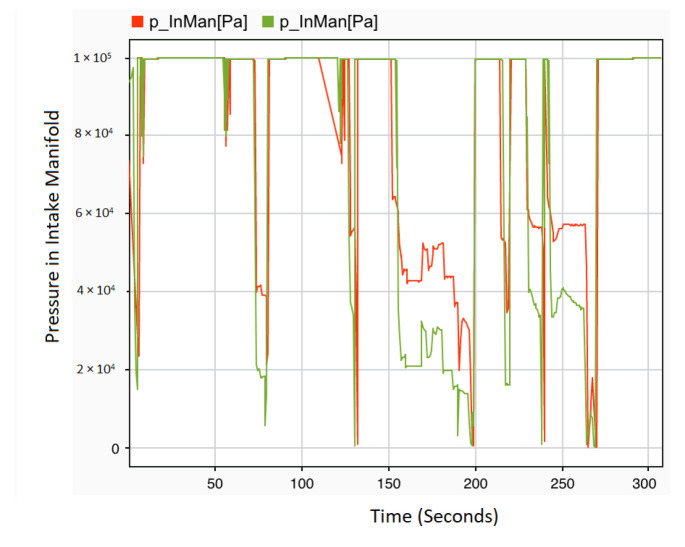
Intake manifold with ignition angle control signal stuck-at fault injection.

On the other hand, it is worth noting that there is no significant impact on the engine speed and vehicle speed caused by the injection of multiple faults in actuator control signals compared to the healthy signals. However, when the stuck-at fault with $d_{v}$ and $o_{v}$ values of 0 (1) is injected into the ignition angle control signal for all of the cylinders at 131 s of the driving scenario, a slight difference is observed in the intake manifold between 155 s to 190 s, as can be seen in Figure 13, where the effect of the stuck-at fault on the pressure in intake manifold signal can be illustrated.

### 5.2. Real-Time Transient Fault Injection

**Figure 14:**
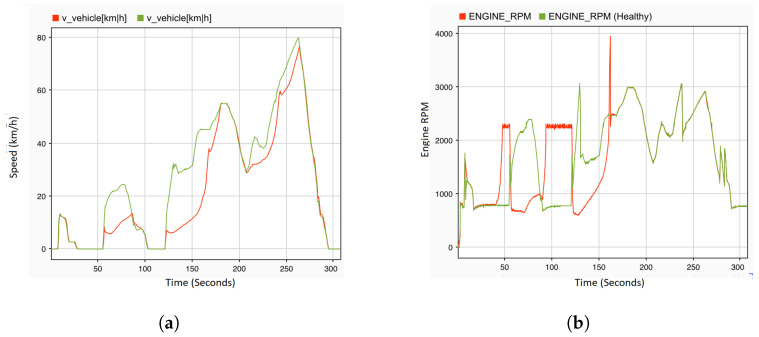
System output: vehicle speed and engine RPM under transient faults injection. (**a**) Vehicle speed sensor with transient stuck-at fault. (**b**) Engine RPM sensor with transient stuck-at fault.

Since transient faults occur much more frequently than permanent faults, with more difficulty in detection, this study highlights the effect of this type of fault on the dynamic behavior of the complex automobile system. To this end, the stuck-at fault with $d_{v}$ and $o_{v}$ values of 0 as a transient fault is injected into the engine speed sensor for a period of 160 s at 40 s. Figure 14 shows the effect of the fault on the system behavior, where the stuck-at fault with $d_{v}$ and $o_{v}$ values of 0 (1) as a transient fault is injected into the engine RPM sensor. The time period of the transient fault is from 40 to 160 s. The result shows that the effect is similar to the effect of a permanent fault. However, when the fault is revoked, a sharp increase in both the engine RPM (Figure 14b) and vehicle speed (Figure 14a) at a time of 160 s is observed. Once the fault is deactivated, the system behavior returns to a normal state and continues with the fault-free mode.

## 6. Conclusions

In this study, a novel real-time FI framework based on a HiL simulation system is proposed. The objective of the framework is to conduct an analysis of the dynamic behavior of complex automotive software systems under abnormal conditions during the V-cycle development process, i.e., system integration phase, taking into account the real-time constraints. The key strength of the framework is its ability to analyze the effect of single and multiple faults on the system behavior in real time for the closed-loop automotive control system. Thanks to these features, the validation of the real/soft ECU performance can be carried out with complex test scenarios under nominal and faulty conditions.

The advantages of the framework presented in this paper are illustrated using a gasoline engine as a case study from the automotive domain. The effects of different types of faults on the output variables of the gasoline engine system, i.e., engine RPM, vehicle speed and intake manifold variables, have been illustrated. From a novelty standpoint, the entire vehicle system models, including the vehicle dynamics model, the engine model, the powertrain model and the environment model, are considered. Additionally, nine different types of faults are included in the proposed work to cover the most common potential faults in automotive software signals, such as gain, offset/bias, noise, hard-over, spike, stuck-at, packet loss, delay and drift faults. Besides, the selected fault types are implemented programmatically in the HiL system without altering the original system model. The applicability of the presented framework is demonstrated by injecting permanent and transient faults into the system during the real-time driving scenario. The results exhibit the capabilities of the proposed framework in analyzing the behavior of complex automotive systems, allowing for all types of faults defined in Table 2 to be injected into the corresponding signals in real time. Moreover, the results also show its ability to enable FI in different locations and at different times individually or simultaneously.

In the future, this work can be extended in two different research directions. On the one hand, artificial intelligence technology, e.g., machine learning, can be used to automatically generate and execute fault test cases based on functional safety requirements. By doing so, instead of a manual random selection of fault configurations, a systematic evaluation of fault effects can be achieved, enabling higher fault coverage and accelerating the prediction of catastrophic fault parameters. On the other hand, the proposed work can be integrated in the process of safety analysis, i.e., in the HARA activities, so that its useful usability in industrial applications can be broadened, especially for the goal of a safety assessment of the SUT in compliance with the ISO 26262 standard. Furthermore, with respect to robustness testing, it can be further developed into a powerful tool for the benchmark validation of fault tolerance mechanisms in autonomous systems. In addition to the aforementioned future directions, the results of sensor and control actuator FI can be used as a dataset for developing an intelligent multi-class fault detection and diagnosis model. To be specific, by collecting accurate faulty and healthy datasets from the vehicle system models as a result of FI, the limitation of the lack of datasets for training and testing fault detection and the diagnosis model can be overcome. 

## Figures and Tables

**Table 1:** Overview of the related works.

Related Works	Approach	Application Domain	Single/ Multiple	Fault Models	Real-Time Constraints	Assessment
Moradi et al. [10]	Model-implemented hybrid FI	FI for cyber–physical systems (CPS)	Single	Six HW fault types	Yes	High manual effort High fault coverage Low fidelity simulations
Saraoglu et al. [41]	Simulation-based FI	FI for autonomous driving systems	Single and Multiple faults	Two fault types	No	Low manual effort Low fault coverage High fidelity simulations
Juez et al. [42]	Simulation-based FI	FI for automotive systems	Single	Two fault types	No	High manual effort Low fault coverage High fidelity simulations
Poon et al. [51]	Simulation-based FI and hardware-based FI	HiL design and testing for electric vehicle drive systems	Single	Three fault types	Yes	Low manual effort Low fault coverage High fidelity simulations
Yang et al. [52]	Signal-conditioning-based FI	FI for traction control system of high speed trains	Single	Three fault types	Yes	High manual effort Low fault coverage High fidelity simulations
Garramiola et al. [53]	Model-implemented FI	Enhanced FMEA for railway traction drive	Single	Three fault types	Yes	High manual effort Low fault coverage High fidelity simulations
Elgharbawy et al. [54]	Run-time-implemented FI	FI for testing the robustness of the fusion algorithms of (ADAS)	Single	Three fault types	Yes	Low manual effort Low fault coverage High fidelity simulations
Zhang et al. [55]	Model-implemented FI	FI for fault diagnosis of induction motor	Single	One fault type	Yes	Low manual effort Low fault coverage High fidelity simulations
Garramiola et al. [56]	Model-implemented FI	Hybrid sensor fault diagnosis in railway traction drives	Single	Two fault types	Yes	High manual effort Low fault coverage High fidelity simulations
Fu et al. [57]	Software-based FI	FI for safety validation of autonomous vehicles	Single	Seven fault types	Yes	Low manual effort High fault coverage High fidelity simulations
Park et al. [58]	Software-based FI	FI for AUTOSAR-based automotive software development	Multiple faults	Five fault types	Yes	Low manual effort High fault coverage Low fidelity simulations
**Proposed FI framework**	**Model-implemented FI & Signal-based FI**	**FI for automotive systems development**	**Single and multiple faults**	**Nine fault types**	Yes	**Low manual effort** **High fault coverage** **High fidelity simulations**

**Table 2:** Value of dv and ov for all fault types.

Fault Type	(dv) Value	(ov) Value
Healthy Signal	1	0
Stuck-at Fault	0	0 or 1, and it varies on time
Offset/Bias Fault	1	fixed constant value
Gain Fault	Greater than 1	0
Noise Fault	1	random value
Hard-Over Fault	0	higher than maximum threshold
Spike Fault	1	value varies on time
Drift Fault	1	value increases on time
Packet Loss Fault	0	0
Delay Time Fault	0	last cycle value of h(t) based on time given

**Table 3:** Selected location of fault occurrence.

Name	Type	Unit
Acceleration Pedal Position	Sensor	[%]
Engine RPM	Sensor	[rpm]
Mass Flow Through Throttle	Sensor	[kg/h]
Fuel Meter Unit	Control Signal for Actuator	[mA]
Pressure Value	Control Signal for Actuator	[mA]
Ignition Angle	Control Signal for Actuator	[rad]
Crank Angle	Control Signal for Actuator	[aTDC]

**Table 4:** Case study parameters.

Parameter Name	Unit	Value
Vehicle mass	[kg]	1250
Dynamic tire radius	[m]	0.35
Maximum brake force	[N]	28,000
Air density	[kg/m${}^{3}$]	1.1842
Rolling resistance coefficient	[-]	0.01
Exhaust manifold volume	[m${}^{3}$]	0.002
Number of engine cylinders	[-]	8
Intake manifold volume	[m${}^{3}$]	0.008
Intake manifold area	[m${}^{2}$]	0.5
Maximum flow area for throttle valve	[m${}^{2}$]	0.0020399
Turbocharger upper limit of compressor pressure	[Pa]	200000
Maximum air mass in cylinder	[kg]	0.00343486
Injection type switch	Direct/Port	Direct
Fuel tank volume	[l]	60
Bulk modulus of gasoline fuel	[bar]	13800
Gasoline fuel density	[kg|m${}^{3}$]	725
Gas constant of air	[J|(kgK)]	287
Gas constant of exhaust	[J|(kgK)]	285
Gas constant of fuel	[J|(kgK)]	75.5861
Piston area	[m${}^{2}$]	0.0029274
Compression ratio	[-]	10.3
Water temperature	[degC]	25
Gain for air cooling with fan	[W|K]	50.2655

**Table 5:** Configuration of permanent single fault injector.

Fault Type	Fault Location	Fault Time	Fault Value
Gain	Acceleration Pedal Position Sensor	5	10
Offset	Engine RPM Sensor	24	1800
Noise	Acceleration Pedal Position Sensor	5	0–100
Packet Loss	Rail Bar Sensor	120	0
Stuck-at	Engine RPM Sensor	5	0
Stuck-at	Ignition Angle Control Signal	131	0
Drift	Acceleration Pedal Position Sensor	30	1
Hard-Over	Acceleration Pedal Position Sensor	36	127
Spike	Mass Flow Through Throttle Sensor	0	1–510
Delay	Engine RPM Sensor	120	5

**Table 6:** Configuration of permanent simultaneous fault injector.

Fault Type	Fault Location	Fault Time	Fault Value
Noise	Acceleration Pedal Position Sensor	25	0–100
Stuck-at	Engine RPM Sensor	35	0

**Table 7:** Configuration of transient fault injector.

Fault Type	Fault Location	Inject Time	Eject Time	Fault Value
Stuck-at	Engine RPM Sensor	40	160	0

## Data Availability

Data available on request due to restrictions.

## Abbreviations

The following abbreviations are used in this manuscript:

HiL	Hardware-In-the-Loop Simulation
ECU	Electronic Control Unit
FI	Fault Injection
FTA	Fault Tree Analysis
FMEA	Failure Mode and Effect Analysis
SUT	System Under Test
ASM	Automotive Simulation Models
CAN	Controller Area Network
V&V	Verification and Validation
